# MEMS Micromirror Actuation Techniques: A Comprehensive Review of Trends, Innovations, and Future Prospects

**DOI:** 10.3390/mi15101233

**Published:** 2024-09-30

**Authors:** Mansoor Ahmad, Mohamed Bahri, Mohamad Sawan

**Affiliations:** 1 CenBRAIN Neurotech, School of Engineering, Westlake University, Hangzhou 310030, China; mansoorahmad@wioe.westlake.edu.cn (M.A.); mohamedbahri@sz.tsinghua.edu.cn (M.B.); 2 Zhejiang Key Laboratory of 3D Micro/Nano Fabrication and Characterization, Westlake Institute for Optoelectronics, Fuyang, Hangzhou 311421, China; 3 Institute of Biopharmaceutics and Health Engineering, Tsinghua Shenzhen International Graduate School, Shenzhen 518055, China

**Keywords:** MEMSs, MEMSs mirrors, microactuators, microelectromechanical systems, micromirrors, optical scanning

## Abstract

Micromirrors have recently emerged as an essential component in optical scanning technology, attracting considerable attention from researchers. Their compact size and versatile capabilities, such as light steering, modulation, and switching, are leading them as potential alternatives to traditional bulky galvanometer scanners. The actuation of these mirrors is critical in determining their performance, as it contributes to factors such as response time, scanning angle, and power consumption. This article aims to provide a thorough exploration of the actuation techniques used to drive micromirrors, describing the fundamental operating principles. The four primary actuation modalities—electrostatic, electrothermal, electromagnetic, and piezoelectric—are thoroughly investigated. Each type of actuator’s operational principles, key advantages, and their limitations are discussed. Additionally, the discussion extends to hybrid micromirror designs that combine two types of actuation in a single device. A total of 208 closely related papers indexed in Web of Science were reviewed. The findings indicate ongoing advancements in the field, particularly in terms of size, controllability, and field of view, making micromirrors ideal candidates for applications in medical imaging, display projections, and optical communication. With a comprehensive overview of micromirror actuation strategies, this manuscript serves as a compelling resource for researchers and engineers aiming to utilize the appropriate type of micromirror in the field of optical scanning technology.

## 1. Introduction

Microelectromechanical systems (MEMSs) mirrors, also known as micromirrors, have recently garnered significant attention in the research community and the optical industry due to their applications in numerous fields. MEMSs micromirrors cover a wide range of applications, including confocal microscopy [1,2], display projectors [3,4,5,6], optical coherence tomography (OCT) [7,8,9,10], light detection and ranging (LiDAR) systems [11,12,13,14], 3D scanners [15,16], optical switches [17,18,19,20], spectroscopy [21,22], and medical imaging [23,24]. MEMSs scanners have successfully replaced the large sized and power-consuming galvanometer scanners in OCT and confocal microscopy. Thus, they offer a compact, cost-effective, and power-efficient alternative for high-speed beam steering.

The performance of MEMS micromirrors primarily depends on the selection of the actuators, the force generated by the particular actuator, and the geometry of the assembly and micromirror itself. Although MEMS micromirrors can be classified based on several parameters like the degree of freedom, type of mirror surface, and actuation, etc. However, the actuation of these mirrors plays a key role, and therefore we will only focus our discussion on the actuation of micromirrors. Like most MEMSs actuators-based devices, micromirrors primarily use four main types of actuators: electrostatic, electrothermal, electromagnetic, and piezoelectric.

Most micromirrors reported to date have primarily used electrostatic actuation. These electrostatically actuated mirrors have small sizes, high scanning speeds, and low power consumption, but they require a large actuation voltage (greater than 100 V) to achieve the desired angle deflection. Many micromirror applications require large deflection angles and durable structures. However, for achieving large deflection angles, electrostatically actuated micromirrors often lack mechanical robustness because the torsion bars must be very thin to maintain a low spring constant, and a low spring constant leads to pull-in instability.

Electromagnetic actuation offers an appealing alternative for achieving large deflections with high-speed scanning and a strong driving force. However, it requires external magnets, making the micromirror assembly relatively bulky. This is because the materials commonly used in MEMSs, such as silicon and silicon dioxide, have very low magnetic susceptibility and cannot develop significant internal magnetization. Other notable issues with electromagnetic actuation are heat dissipation, high power consumption, and electromagnetic interference.

Electrothermally actuated micromirrors offer a large scan range, strong drive force, low driving voltage, and have no electromagnetic interference or electrostatic discharging issues. However, they tend to consume more power, have a slow response, and face heat dissipation issues. Furthermore, the response speed of electrothermal micromirrors is constrained by thermal expansion, leading to a lower scanning frequency.

The latest addition is piezoelectric micromirrors, which have low power consumption and only need moderately high driving voltage. However, piezoelectric micromirrors not only involve a complex manufacturing process due to the deposition of additional piezoelectric material, but they also have a limited scanning range and a large footprint with a comparatively small reflective surface.

**Figure 1:**
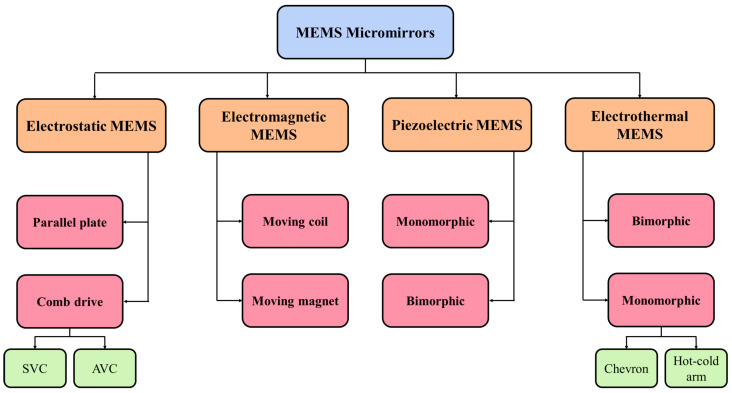
Block diagram of MEMSs micromirror actuation methods.

In short, each type of actuator has its own set of advantages and disadvantages. Several reviews have been reported on micromirrors, each concentrating on different aspects such as a single actuation technique [25], micromirror arrays [26], the material used [27], laser scanning [28], or a particular application like LiDAR [11,12], medical application [9,29,30,31], and smart windows [32]. However, a comprehensive overview that briefly covers all the actuation techniques to provide a broad understanding before selecting the most suitable one has not been addressed previously. This review aims to fill that gap by providing a concise yet detailed exploration of the various actuation methods for MEMS micromirrors. All the key actuation methods adopted in MEMS micromirrors to be discussed are summarised in Figure 1. The actuation principle of each technique, along with their advantages and disadvantages, will be discussed. Furthermore, different mechanical designs reported to achieve the actuation of these mirrors are covered.

The rest of the paper is organized as follows: Section 2 discusses electrostatically actuated micromirrors, which are further divided into parallel plate actuators and combdrive actuators. Section 3 focuses on electrothermal actuation-based micromirrors. Section 4 and Section 5 describe electromagnetic and piezoelectric actuators-based micromirrors, respectively. Section 6 summarizes the hybrid micromirrors, which combine two different types of actuators into a single device.

## 2. Electrostatic Actuation

Electrostatic actuation (ESA) is among the most widely utilized methods in MEMS micromirrors. ESA has the oldest history of implementation in micromirrors, where the movement of microstructures is induced by the application of electrostatic forces. These forces arise due to the attraction or repulsion between charged components, typically electrodes and movable mirrors, creating a displacement that results in the desired mechanical motion. ESAs are preferred because of their ability to be embedded within a chip, allowing for seamless integration with other components. These actuators induce a relatively low magnitude of the electrostatic force, resulting in low power consumption.

Since these devices form a capacitive structure, capacitive loads do not induce steady-state currents, which theoretically eliminates concerns about heat dissipation and potential degradation of device reliability. Despite these advantages, ESAs also have some limitations. The electrostatic force is inherently nonlinear, which can lead to instability and pull-in phenomena, where the movable mirror plate is attracted towards the stationary electrode beyond a certain voltage. This can limit the available range of motion. Additionally, the fabrication of ESA-based micromirrors requires precise control over the gap between the movable mirror and the stationary electrodes, which can be challenging.

Electrostatic actuators can be classified into two main categories based on the dominant factor that increases the tilt angle: variable gap type actuators and variable area actuators. The variable gap type is commonly known as a parallel plate actuator. The variable area type, known as a combdrive actuator (CDA), features numerous parallel electrode fingers resembling a comb. One notable characteristic of electrostatic actuation, particularly in parallel plate actuators, is the pull-in or pull-out effect. This phenomenon occurs when the electrostatic force increases rapidly enough to surpass the mechanical restoring force, resulting the movable plate to be attracted or repelled by the fixed plate. A parallel plate actuator consists of parallel plates separated by an air gap, which act as electrodes and accumulate static charge. These actuators operate by exploiting variations in the gap between the plates. The rotatable top plate is either connected to a torsional elastic beam or bending beams with restoring stroke force, while the fixed plate remains stationary.

Similar to parallel plate actuators, CDAs also comprise movable and stationary parts, taking an interdigitated arrangement that forms the combs. A CDA also possesses high force density, which helps in lowering the actuation voltage. The deflection and displacement in the moving comb are generated by Coulomb forces. Electrostatic actuators typically fall into two categories depending on their movement orientation: in-plane and out-of-plane. For CDAs, the actuators that move laterally in-plane are known as lateral CDAs or the staggered vertical comb (SVC) actuators, while those moving in the out-of-plane direction are referred to as vertical CDAs or angular vertical comb (AVC) actuators. In these actuators, the deflecting mirror can be separated from the actuating component, enabling for a larger deflection of the mirror.

Both parallel plate and combdrive micromirrors offer distinct advantages and are employed in various applications based on their specific design characteristics. Parallel plate micromirrors are favored for their simplicity, low power consumption, and ease of fabrication, making them suitable for compact optical systems and beam-steering applications. On the other hand, combdrive micromirrors excel in applications requiring high-resolution imaging, precise laser scanning, and optical switching, thanks to their capability for larger deflection angles and improved optical performance.

The actuation approaches incorporated by both ESA methods will be further discussed in the following sections.

### 2.1. Parallel Plate ESA-Based Micromirrors

Parallel plate micromirrors represent a basic yet effective design in electrostatic MEMS technology. They consist of a reflective mirror suspended between two parallel electrodes. When a voltage is applied across the electrodes, it generates an electrostatic force, causing the mirror to tilt or rotate. This movement enables precise control of the reflected light beam, making parallel plate micromirrors suitable for applications requiring simple actuation mechanisms and moderate angular deflections.

**Figure 2:**
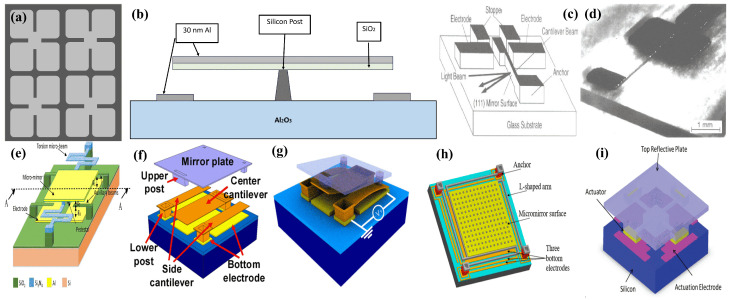
(**a**) Illustration of the first ESA micromirror exhibiting four-leaf clover shape; (**b**) cross-sectional view of single element. Lateral motion micromirror: (**c**) schematic diagram; (**d**) optical photo of the fabricated device. Adapted with permission from Ref. [34]. Copyright 1995, IOP Science. (**e**) Micromirror design with pedestal and auxiliary fixed-free beams. Adapted with permission from Ref. [45]. Copyright 2018, Springer Nature.; Bending arm based micromirrors: (**f**) schematic diagram of interdigitated cantilevers; (**g**) operational mechanism. Adapted with permission from Ref. [46]. Copyright 2009, IEEE. (**h**) L-shaped arm based micromirror. Adapted from Ref. [47], Available under a CC-BY license. Copyright 2020, MDPI. (**i**) Double-bridge micromirror. Adapted with permission from Ref. [21]. Copyright 2020, IEEE.

The first electrostatically actuated micromirror was reported in 1975 by Guldberg et al. [3,33]. When activated by an electron beam, the presented ESA-based mirror array experiences electrostatic deflection towards the static substrate. The micromirror is made up of a silicon dioxide ($SiO_{2}$) membrane grown on epitaxial silicon (Si) sapphire, with aluminum (Al) electrodes structured on top. The release of $SiO_{2}$ layers is achieved through the underetching of silicon, forming the deflectable mirror surfaces. Figure 2a,b illustrate the mirror exhibiting the four-leaf clover shape, along with its corresponding cross-section.

With the progress of MEMS technology, micromirrors gained much more attention around the beginning of the 21st century [34,35,36,37,38,39,40]. Uenishi et al. [34] presented a micromirror demonstrating lateral movement under electrostatic force, as illustrated in Figure 2c,d. The demonstration of this device has opened a window to realize chip-integrated miniaturized optical systems, such as interferometers, improving the reliability and performance of these systems.

In 1996, Chung et al. [41] introduced a vertical motion mirror based on electrostatic torsional forces. The 10 μm × 10 μm aluminum mirror is supported by nickel posts with a gap of 10 μm between the substrate and the mirror. The maximum deflection of the mirror (10 μm) occurs at 35 $V_{dc}$. In another study, Bulher et al. [42] used aluminum for the posts, as well as the mirror plate. The mirror, sizing 30 μm × 40 μm, can tilt by 0.3 μm and cover an angle of $3.2^{\circ }$ when a voltage of 14 V is applied. A number of efforts have been made to improve torsional forces based micromirrors for a high aspect ratio [43] and motion linearity [44], among others.

The major concerns with ESA micromirrors are the requirement of high operational voltages and pull-in instability, which causes a limit to the stroke and rotational angles. A high voltage is required to stage a large driving force for substantial angular displacement. However, elevating the applied voltage results in decreasing the stiffness of the mechanical design leads to destabilization of the system by experiencing a saddle-node bifurcation. As an effort to reduce the driving voltage, Yoon et al. [48] presented a two-electrode ESA system with a trench structure. The two electrodes are positioned horizontally and vertically to facilitate extensive rotational movement. One electrode is placed at the bottom, whereas the second is placed along the vertical sidewall. The vertical electrode serves a dual purpose: stabilizing mirror movement and enhancing electrostatic force. The system can achieve a 1D $90^{\circ }$ rotational angle when actuated at 47 V. A 2D scanning mirror, having a 1 mm^2^ surface with sidewall actuators, was later reported by Bai et al. [49,50] in an effort to replace the galvoscanner in MACROscopes. Spring-shaped torsion beams were also introduced in an effort to minimize the pull-in voltage, which achieved an angular deflection of $2^{\circ }$ [51,52]. Afrang et al. [45] introduced pedestal and auxiliary fixed-free beams into their design to increase the stiffness, which are depicted in Figure 2e. The increased stiffness leads to an increase in the rotation angle range by eliminating the pull-in instability. The presented micromirror has a size of 25 × 25 μm^2^ and a rotational scan range of $\pm 12^{\circ }$ at 36.5 V.

Micromirrors are generally fabricated using bulk micromachining with an SOI wafer. Kunnyun et al. [53] proposed an anodic bonding-based technique to a fabricated torsion micromirror, eliminating the need for an SOI wafer. The 20 μm thick mirror, with a size of 610 × 400 μm^2^, can scan an angle of $\pm 1.1^{\circ }$ when a 520 V signal is applied. Another report presented a heavily doped silicon mirror bonded to a conductive polymer actuator via an elastomeric universal joint [54]. This device utilizes two different materials to attain a highly reflective single-crystal silicon surface for the mirror and an extremely flexible elastomer for the universal joint.

All the mirrors discussed above are one-dimensional (1D). Two-dimensional (2D) micromirrors have also been reported for different applications, such as optical coherence tomography (OCT) [55,56], optical switches [57,58], optical scanning [59], and projection displays [60]. Hoa et al. [61] developed a process to devise a 2D torsion micromirror using bulk micromachining. The reported micromirror successfully attained static rotation angles of $\pm 3^{\circ }$ on both axes when actauted at 40 V.

In 2009, Kim et al. [46] reported a new type of ESA called interdigitated cantilevers. The double bridge actuator-based micromirror works on the principal of bending, unlike the previously reported twisting torsional actuators. The mirror, with hidden cantilevers underneath, as shown in Figure 2f,g, has a size of 16×16μm^2^ and can rotate at an angle up to $\pm 10^{\circ }$ with an applied voltage of 54 V. Another micromirror design reports an L-shaped fixture-based micromirror system that can achieve a large stroke while avoiding the electrostatic pull-in effect [62,63]. The proposed design uses a parallel plate mirror with L-shaped arms extending to all four sides, as shown in Figure 2h. The device’s three-bottom electrode configuration creates an upward electrostatic force, unlike traditional mirror systems that have downward attractive forces. As a result, the upward force diminishes the pull-in instability observed in other ESA-based micromirror systems. The reported 400×400μm^2^ polysilicon-based mirror exhibited a 1.65 μm stroke at 100 V [62]. The same approach was later adopted by Aryal et al. [47] to enhance the stroke to 5 μm when a 150 $V_{dc}$ signal is applied. Wang et al. [64] proposed a verifocal mirror supported by four beams at the sides. The mirror takes a concave shape when the beams are actuated and thus can focus the light from 79.8 mm to 2.03 mm at different voltages. In 2020, Kumar et al. [21,22,65] proposed another double-bridge micromirror with a simplified fabrication process and improved the pull-in voltage. The mirror consisting of a substrate, cantilevers, and mirror plate layers, is shown in Figure 2i. The micromirror capable of tip-tilt-piston (TTP) movement had a size of 200×200μm^2^ and achieved a rotational angle of $\pm 1.5^{\circ }$ at only 23.1 V.

### 2.2. Combdrive ESA

Combdrive-actuated micromirrors are the more sophisticated variation of electrostatic MEMSs devices, offering enhanced performance and versatility. In combdrive configurations, the movable mirror is attached to a set of comb-like structures with interleaved fingers connected to fixed and movable electrodes. An electrostatic force is generated by applying a voltage between these electrodes, causing the combdrive structure to move. This movement results in the tilting or rotation of the mirror, enabling relatively larger deflection angles and finer control over the reflected light beam. Additionally, the size of the reflective surface can be precisely determined, as the actuators are separated from the reflective mirror. This separation also enables the removal of the substrate under the reflective surface, which helps reduce squeeze-film air damping effect, that can limit the rotation or rotational speed and settling time.

**Figure 3:**
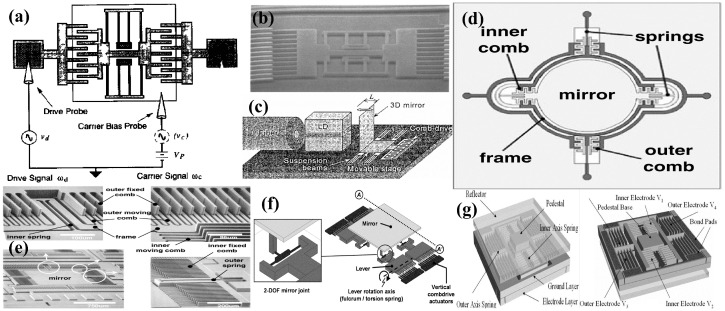
Electrostatic combdrive: (**a**) schematic diagram with electrical connections; (**b**) SEM image of combdrive connected to linear resonant plate. Adapted with permission from Ref. [66]. Copyright 1990, Elsevier. (**c**) 3D polysilicon micromirror-based tunable laser diode. Adapted with permission from Ref. [68]. Copyright 2001, IEEE. 2D vertical combdrive micromirrors: (**d**) Schematic diagram; (**e**) SEM image with some magnified parts. Adapted with permission from Ref. [71]. Copyright 2004, IOP Science. (**f**) Schematic structure of the motion-amplifying levers based two-axis mirror. Adapted with permission from Ref. [72]. Copyright 2006, IEEE. (**g**) (**left**): High fill factor micromirror illustration with transparent mirror for better visualization. (**right**): Bonding pads to connect to the electrodes. Adapted with permission from Ref. [73]. Copyright 2006, IEEE.

A combdrive actuator (CDA) for optical system applications was proposed by Tang et al. in 1990 [66]. The geometrical concept and an image of the fabricated polysilicon based combdrive is shown in Figure 3a,b. In 1998m Juan et al. [17] and Kiang et al. [67], in two separate studies, reported the utilization of vertical combdrives in micromirrors for optical scanning to scan optical beams parallel to the substrate. In vertical combdrives, the fixed and movable teethes are staged in the vertical direction. Later, a scanner integrated along the substrate, comprising a polysilicon-based 3D micromirror, shown in Figure 3c, was successfully integrated into a tenable laser diode system [68]. Vertical CDAs were also utilized for mirror movements against the plane of the substrate [69]. The reported CDA micromirror was fabricated on a movable upper comb teeth array in the upper device layer, connected to the anchored torsional springs, whereas the static bottom comb is fabricated in a different layer. A voltage applied across the upper and lower comb arrays generates a vertical electrostatic force, resulting in either torsional or piston-style motion of the micromirror. This structure was employed in a 1D micromirror with a 450 μm diameter exhibiting a physical tilt angle of $\pm 1^{\circ }$ at 100 $V_{dc}$ [70].

Dual-axis vertical CDA systems were also reported to achieve 2D optical scanning [71,74,75]. Hah et al. [71] presented 2D micromirrors adopting a gimbal configuration, which consist of an inner and an outer frame. Inner torsion springs connect the mirror to the inner frame, which in turn connects to the outer frame or substrate, as shown in Figure 3d,e. The 2D scan micromirror, sized at 1 mm × 1 mm, can achieve a mechanical scan angle of $\pm 1.8^{\circ }$ on the outer axis and up to $\pm 2.1^{{}^{\circ}}$ on the inner axis when biased at 48 V. In another report, a 1.5 mm × 1.5 mm micromirror achieved a tilt of $\pm 15^{\circ }$ when excited at 40 $V_{dc}$[74]. Additionally, a system comprising a micromirror sized at 650×650μm^2^ and driven by 20 $V_{DC}$ and $15V_{pp}$ attained scanning angles of $27^{\circ }$ & $20^{\circ }$ [75].

A fill factor that defines the ratio of the reflecting surface to the total size of the device is an important factor in optical scanning systems. However, CDA-based micromirror systems generally often suffer from low fill factors. In this regard, Tsai et al. [72,76] demonstrated a CDA analog micromirror design, as shown in Figure 3f, which incorporates a motion-amplifying levers and offers a high fill factor of 98%. The micromirror can scan through a mechanical angle, the actual angular displacement of the device itself during actuation, of $\pm 1.8^{\circ }$ on both axes at 75 V. The micromirror also demonstrated a piston motion of 11.7 μm when all actuators were biased simultanously.

Jung et al. [73] presented a micromirror array with high fill factor, exhibiting TTP movements, and it is depicted in Figure 3g. SOI-based processing was used to fabricate the actuator and reflector on one wafer, and separate thin-film processed wafers were used for electrical wiring. Subsequently, flip-chip bonding was used to bond electrical wiring to the actuator/reflector chip, thus electrically addressing the array. The mirror array has a pixel size of 360 μm, achieving optically flat reflectors with a high fill factor of 99%.

Researchers are currently focused on improving the design [77,78,79,80], application [1,2,81,82,83], and control [84,85] of MEMS actuators for micromirror systems. Achieving a large rotation angle and high vertical movement is one such characteristic being targeted [86,87,88]. Another important aspect is the settling or shifting time. The actuators often exhibit underdamping, as adjusting damping in MEMS design is often ignored due to additional complexities. Consequently, this underdamping leads to residual vibrations, increased overshoot, and extended settling times. One way to reduce the settling time is the use of input shaping [89]. Input shaping is a used method to suppress the residual vibration to reduce the settling time without the use of an additional feedback component. Another important parameter is the operation voltage. It has been reported that reducing the ambient pressure can reduce the fractional loss, and this consequently results in reduced operational voltage [4]. One such effort to reduce the operational voltage by reducing the pressure was made by [90]; however, the pressure was not reduced enough. A 1D combdrive micromirror attaining high vacuum was demonstrated by Chu et al. [91] by employing a transistor outline-8 metal can package. The same study was then extended further to exhibit a 2D laser scanning with a voltage decreased by 32 folds compared to the voltage at atmospheric pressure [92].

**Figure 4:**
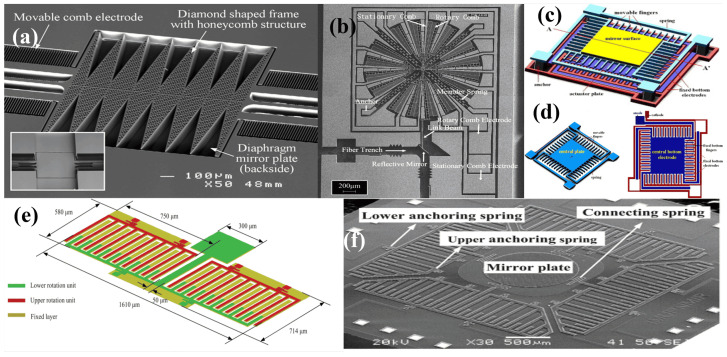
A 2D vertical combdrive micromirror: (**a**) SEM images of the diamond shaped diaphragm. The mirrored surface of the device is shown in the bottom left small image. Adapted with permission from Ref. [93]. Copyright 2006, IOP Science. (**b**) VOA device using rotary CDA. Adapted with permission from Ref. [95]. Copyright 2006, IEEE. (**c**) Schematic of the repulsive actuators micromirror. (**d**) top and bottom plates. Adapted with permission from Ref. [96]. Copyright 2012, Elsevier. (**e**) Schematic of two-row repulsive torque single actuator. (**f**) SEM image of fabricated micromirror. Adapted with permission from Ref. [60]. Copyright 2015, IEEE.

In raster scanning displays, the shape and size of micromirrors significantly impact the resolution and scan speed. Larger mirrors offer higher resolution but also increase mass. Thicker plates reduce deformation during actuation, improving beam quality. However, thick and heavy plates result in higher driving voltages and reduced scan speed. To address these challenges, Ji et al. [93] presented a scanning micromirror with rhombic-shaped frames, as shown in Figure 4a. This design features a diaphragm mirror plate with dimensions of 1.5×1.5 mm^2^ supported by 110 μm thick, lightweight rhombic-shaped frames. A $8.5^{\circ }$ mechanical deflection angle was achieved while aiming for faster scanning and improved optical performance. Another effort in this regard was made by integrating carbon nanotube (CNT) hinges into the actuators [94]. Since CNTs have low resistivity, they were employed both as mechanical structures and the electrical interconnects, eliminating the need for an additional electrical metal layer. The CNT-integrated actuator micromirror achieved a tilt angle of $1.5^{\circ }$ at 5 V.

Mirror actuators are generally engineered to either move along a specific axis, rotate against torsion springs, or tilt using pivots. However, for some specific applications, such as variable optical attenuators (VOAs), a rotational tilt is desired, as it offers better attenuation performance [97]. In this regard, Yeh et al. [95,98] presented a planner rotational tilt micromirror driven by a rotatory CDA to achieve a wide attenuation range. In the presented device, the CDA is attached to a planer mirror tilted at $45^{\circ }$, as depicted in Figure 4b. The design demonstrated an attenuation range of 57 dB while rotating up to $2^{\circ }$ under $4.5\;V_{dc}$. In 2018, Cheng et al. [99] presented a vertical VOA attenuator with $0.98^{\circ }$ rotation at $8\;V_{dc}$, achieving an attenuation range of 55 dB.

Electrostatic attractive actuators possess a "pull-in" instability effect, which in turn confines the reliability and stroke of the micromirrors. To address this issue, repulsive actuators have been introduced, which effectively eliminate the pull-in instability issues [100,101]. However, these devices typically have a low fill factor, affecting the compactness of the whole device. Subsequently, Hu et al. [96] employed the same concept to present a low-voltage repulsive actuator micromirror. The micromirror, depicted in Figure 4c,d, has a size of 190×190μm^2^ and can reach a displacement of 1.2 μm at 60 V. Fan et al. [60] presented a double-row CDA to induce out-of-plane rotation to rule out pull-in instability and avoid the limitation posed by the gap between the substrate and moving fingers. The nonlinearly actuated micromirror, shown in Figure 4e,f, with a 1 mm reflective diameter, achieved a mechanical rotation of 11.5 μm when actuated at 60 V. Another approach to minimize the pull in instability is to adjust the tensile stress in the torsion beam to introduce nonlinearity in the spring effect [102]. In a most recent study, Shan et al. [103] thoroughly investigated the conditions leading to pull-in instability and analyzed methods to refine them. Various instability models were explored to understand the factors causing instability and determine stable driving voltages for smooth operation.

## 3. Electrothermal Actuation-Based Micromirrors

In thermal actuation, electrical energy is transformed into mechanical energy. The thermal actuation mechanism relies on the thermal expansion of solids, fluids, and gases. Thus, there are three types of thermal actuation: solid, liquid, and gas. The solid-based thermal actuation can be further categorized into a single-material solid (monomorph), bimaterial solid (bimorph), and multi-material solid (multimorph).

The single-material solid thermal actuator comprises a single material having different thicknesses at the ends. The thin end has more resistance than the thick one, and hence the temperature at the narrow end becomes higher. Consequently, different thermal expansions take place in the narrow and wide regions, which eventually lead to a deflection in the beam. Another way to construct thermal actuators is to stack together two materials having different thermal expansion coefficients (TECs), which is termed a bimorph thermal actuator. The material with higher TECs expands more when there is a temperature rise compared to the one with lower TECs, thus inducing a deflection in the bimorph by bending towards the material having lower TECs.

Liquid thermal actuation is based on the deformation caused by changes in surface tension with temperature variations. Gas-based thermal actuation is based on a gas-filled sealed cavity inducing thermopneumatic actuation. Tang et al. [25] discussed thermal actuation effects and electrothermal actuation mechanisms in details. Here, we will focus on the utilization of these mechanisms in MEMS micromirrors. Also, though fluidic thermopneumatic actuation-based micromirrors have been reported in the literature [104,105,106,107]. However they have not garnered significant interest among researchers; therefore, our discussion will be confined only to solid-based thermal actuation.

### 3.1. Monomorphic Electrothermal Actuators

**Figure 5:**
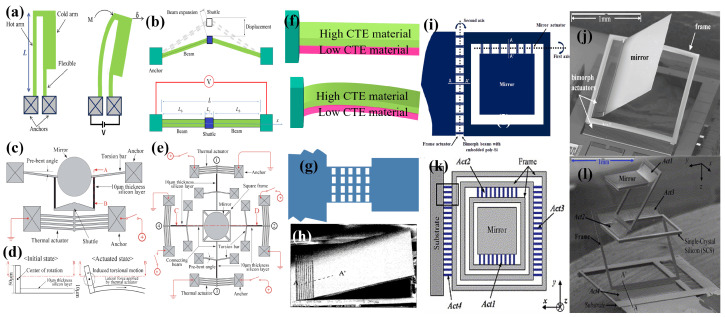
Monomorphic electrothermal actuators: (**a**) Configuration of a U-shaped actuator. (**b**) Illustration of Chevron electrothermal actuator. (**c**) A 1D Chevron actuator micromirror. Presented by [108]. (**d**) Conversion of lateral motion to torsional motion. (**e**) A 2D micromirror actuator. Adapted with permission from Ref. [108]. Copyright 2009, IOP Science. (**f**) Illustration of a bimorphic contilever in pre-actuation and under-actuation state. (**g**) conceptual diagram of the 1D micromirror presented by [109] and its (**h**) SEM image. Adapted with permission from Ref. [109]. Copyright 2002, IEEE. (**i**) Illustration of 2D micromirror proposed by [110]. (**j**) SEM image of the fabricated 2D micromirror.Adapted with permission from Ref. [110]. Copyright 2004, IEEE. (**k**) Top view of the paired actuators mirror by [111]. (**l**) SEM image of the fabricated mirror. Adapted with permission from Ref. [111]. Copyright 2006, Elsevier.

The most common and simple single-material electrothermal actuator is a U-shaped actuator. Also known as a hot–cold arm actuator, it consists of two arms with different thicknesses. This difference in thickness results in the generation of more heat in the thinner arm, leading to different thermal expansions in each arm. As a result, a deflection is induced in the attached microstructures. The design configuration of the U-shaped thermal actuator and its bending upon applying an electric signal is illustrated in Figure 5a.

The U-shaped actuation method in micromirrors was first adopted by JR.Reid et al. in 1996 [112,113]. An array of actuators was used to generate a significant amount of force to drive the micromirror, enabling to manipulate optical beams over a range $15^{\circ }$. Later, JR.Reid et al. [114] presented another hot–cold arm actuator array designed to vertically lift the micromirror system. However, hot–cold actuator-based micromirrors have not been reported subsequently. This may be because of the inability of the actuators to induce deflection in both inwards and outwards deflection.

Another category of single-material micromirrors is known as Chevron electrothermal actuators. The most common Chevron actuators are V-shaped, as illustrated in Figure 5b. However other shapes, such as Z-shaped, have also been reported [115]. A Chevron actuator consists of a pair of slanted beams connected to a shuttle and fixed at the ends. Typically, an array of beams is connected to a single shuttle. When an electric current passes through the beams, resistive heat is generated, which induces thermal expansion. An inclination in the beam takes place because of the thermal expansion, which consequently pushes or pulls the shuttle to move in a forward or backward direction [116].

Chevron ETAs have also been used in optical switching and MEMS mirror systems [20,108]. Eun et al. [108] presented a V-shaped actuation mechanism to induce one- and two-directional motion in micromirrors. The 1D actuation system, comprising a single V-shaped ETA, as shown in Figure 5c–e, can be driven at up to 13 V to cover a scan angle of $6.5^{\circ }$, whereas 2D micromirrors consisting of four V-shaped ETAs can achieve maximum angles of $5.4^{\circ }$ and $5.2^{\circ }$ at 11 $V_{dc}$.

The monomorphic ETAs generate small forces, and usually, an array of actuators is required to produce sufficient force for lifting micromirrors. Consequently, the micromirrors have a small fill factor. This may be a reason that the researchers have not been very drawn to them.

### 3.2. Bimorphic Electrothermal Actuators

Bimorphic ETAs are made by layering two materials with different TECs on top of each other. One material has a high TEC, whereas the other has a low TEC. The deflection of the actuator relies on the difference in the TEC, the geometry of the beam, and the thickness ratio of the materials. During actuation, joule heating induces strain that causes the high-TEC layer to expand more than the low-TEC layer, bending the entire structure out-of-plane or in-plane in the direction of the low-TEC layer. The typical bimorph actuator in its pre-actuation and actuation state is shown in Figure 5f.

The bimorphic ETA was first applied to micromirror actuation in the early 1990s using a bimorph based on Si and Al [117]. Later, a $SiO_{2}$/Al-based bimorph with embedded polysilicon in the oxide for heating was proposed by Buhler et al. [118], achieving the deflection of $4.8^{\circ }$ while consuming a power of 4.8 mW. Only two bimorphs were used in the aforementioned micromirrors, which cannot support mirror sizes larger than a few hundred micrometers. To address this issue, H. Xie et al. [109,119] introduced a $SiO_{2}$/Al bimorphic array-based 1D single-crystalline silicon (SCS) micromirror capable of supporting a mirror size of 1 mm × 1 mm. The reported mirror is shown in Figure 5g,h. An optical scanning angle of around $17^{\circ }$ was achieved with a power consumption of 10 mW.

In 2004, Jain et al. [110] proposed a 2D micromirror, which opened up possibilities for the ETA to be used in optical coherence tomography and other 3D imaging applications, which is only possible with 2D scanning. The 2D movement is achieved by attaching the micromirror to a movable frame through mirror actuators. The movable frame is then attached to the substrate via another identical beam. The device, having an active region of 1 mm^2^, is illustrated in Figure 5i. The bimorph is formed by $SiO_{2}$/Al, with polysilicon buried inside the $SiO_{2}$ for heating the silicon dioxide layer. The mirror can scan up to $40^{\circ }$ in 2D when 95 mW dc power is applied. However, as can be seen in Figure 5j, the mirror has an undesirable tilt of $42^{\circ }$ when released, and the frame tilts up to $16^{\circ }$. Later, Jain et al. [111], proposed a solution to minimize the unwanted tilt by introducing a set of two actuators, as shown in Figure 5k,l. The reported micromirror not only has a reduced the initial tilt, but it can also achieve a vertical displacement of 500 μm when a DC voltage of 15 V is applied. The mirror has a scanning angle greater than $\pm 30^{\circ }$ at 12 V and can cover a $14^{\circ }\times 50^{\circ }$ angular region during raster scanning. In 2007, Wu et al. [120] presented a platinum-based bimorph ETA mirror. Pt was utilized as the heating element and positioned at the end of the bimorph rather than being embedded, as seen with polysilicon. The 1D micromirror using the mesh of the actuators structure can rotate up to $124^{\circ }$ at 12.5 $V_{dc}$.

**Figure 6:**
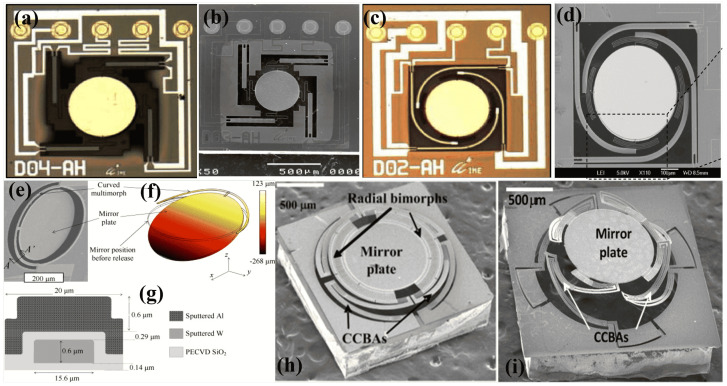
(**a**) Optical image of the micromirror employing linear actuator (**b**) SEM image of the fabricated device. (**c**) Image of the micromirror with curved actuator. (**d**) Corresponding SEM photographs of the device. Adapted with permission from Ref. [24]. Copyright 2007, IOP Science. (**e**) SEM image depicting a circular micromirror. (**f**) Simulated micromirror with initial tilt upon release. The color bar illustrates the out−of−plane displacement from its unreleased position. (**g**) Cross-section illustration of the multimorph actuator. Adapted with permission from Ref. [124]. Copyright 2011, Elsevier. (**h**) SEM image of piston only micromirror presented by [125]. (**i**) SEM image of TTP micromirror. Adapted with permission from Ref. [125]. Copyright 2012, Elsevier.

An alternative method to reduce the initial tilt has been to use curved beam actuators and spiral flexure connectors [24] as Illustrated in Figure 6a–d. This design eliminates dead space on the chip by increasing the length of the actuators through their curved shape while reducing the overall chip size. Additionally, the spiral flexure can be utilized to adjust the initial tilt of the bimorph beam. Some more micromirrors with improved design have also been reported for potentially integrating into OCT systems [121,122,123]. Nevertheless, the utilization of spiral flexures is susceptible to shocks and acceleration, making it an unreliable solution in terms of robustness.

In 2011, Pal et al. [124] demonstrated a semi-circle single-bimorph-based micromirror device, exhibiting a relatively low central shift while maintaining robustness. The micromirror, depicted in Figure 6e,g, features actuators comprising Al/W beams capable of attaining 2D scanning, covering a $60^{\circ }$ angle with an applied voltage 0.68 V and input power of 11 mW. Subsequently, the same group improved the design to achieve TTP and piston-only movements as well [125]. The devised micromirror device consists of three concentric AL/W bimorph actuators, as shown in Figure 6h,i. For the piston-only device, shown in Figure 6h, a piston motion of 200 μm was attained at 0.9 V, with a lateral shift of less than 3 μm. In the case of TTP, shown in Figure 6i, scanning angles of up to $\pm \;11^{\circ }$ were achieved at 0.6 V, with a piston motion of 227 μm and a lateral shift of only 7 μm at 0.8 V.

**Figure 7:**
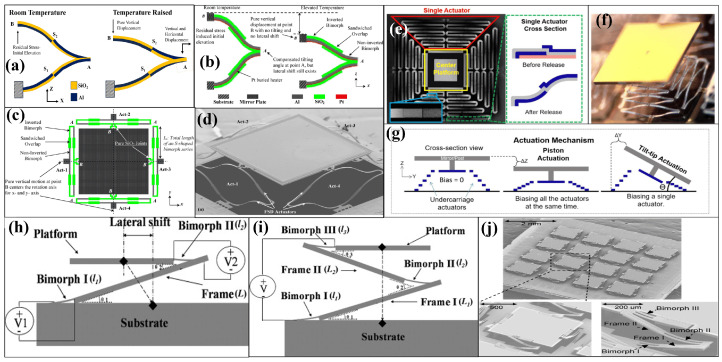
Inverted series connector bimorph (**a**) Concept of ISCB. (**b**) An illustration of the modified ISCB showing the overlapped region. (**c**) Top view of the device design. (**d**) SEM image of the fabricated mirror. Adapted with permission from Ref. [127]. Copyright 2009, IEEE. Three stacked FDS bimorphs proposed by Ref. [129]. (**e**) Composite image of the actuation mechanism. (**f**) Optical image of the final device. (**g**) The actuation motions of the proposed device. Adapted from Ref. [129]. Available under a CC-BY license. Copyright 2021, MDPI. Concept of LSF actuation (**h**) Two bimorph displays LVD with lateral shift. (**i**) A three-bimorph system demonstrates LVD without lateral shift. Adapted with permission from Ref. [130]. Copyright 2008, Elsevier. (**j**) SEM image of the fabricated array device with a detailed single cell and single actuator. Adapted with permission from Ref. [131]. Copyright 2010, IEEE.

Single-bimorph meshed-type array actuators suffer from the thermal coupling and multidimensional motion of the single bimorph. One way to minimize these issues is to use an inverted series-connected bimorph (ISCB), which are commonly known as double S-shaped bimorph actuators [126]. The S-shaped part is composed of two bimorph beams, made of Al and polysilicon-embedded $SiO_{2}$, connected in series, where the adjacent layers have opposite material compositions. In contrast, the inverted series-connected bimorph comprises two S-shaped bimorphs, which are attached together in inverted form as depicted in Figure 7a. The alternating layer construction and double S-shape design ensure that each adjacent bimorph section has equal but opposite curvature upon activation. This allows the entire beam to deform without rotation, resulting in pure displacement in one direction at the tip. The lateral displacement of each S-shape section results in a combined z displacement at a specific point, which is achieved by offsetting the lateral shifts of S1 and S2.

The proposed micromirror is capable of both piston and tip/tilt (TTP) movements. However, the experimental results were not presented due to the loss of electrical connections in the lift-off process. In 2009, Jia et al. [127] adopted the ISCB concept and proposed a modified folded double S-shaped (FSD) bimorph. The modified actuator has an overlap portion between the two S sections, with increased width at the overlapping region, as shown in Figure 7b. The bimorph, composed of Al/$SiO_{2}$, uses Pt instead of polysilicon as the heating material. The topology of the FSD-based device is shown in Figure 7c, and an SEM image of the fabricated device is depicted in Figure 7d. The device demonstrates a three-degree-of-freedom (3-DOF) capability, exhibiting an optical scan range of $\pm 30^{\circ }$ along both the x and y axes and a displacement of 480 μm along the z axis. These movements are achieved using DC voltages below 8 V. Later, in 2011, Jia et al. [128] successfully converted the above-mentioned device into a 4×4 micromirror array device with a high fill factor of 88%.

The FDS bimorph-based micromirror devices has captured significant interest from the community in the years to come [13,132,133]. Samuelson et al. [134] introduced a stacked FDS bimorph for linear lateral motion with ultra-low tilt applications. The stacked FDS bimorph micromirror achieved a highly linear motion of 90 μm with just 1.2 V and ultra-low maximum tilt of $0.25^{\circ }$. Duan et al. [135] utilized the FDS bimorph actuators in a pre-set tilted micromirror for OCT applications. The pre-tilt was achieved by using an additional set of bimorph flexure pairs, achieving a 2D scanning angle of $\pm 40^{\circ }$ at 5.5 V. Later, Tanguy et al. [136] presented a similar device for OCT with a modified configuration of FDS actuators. The actuators were arranged in both series and parallel, forming a mesh structure. This design aimed to enhance the displacement and force of the actuators while maintaining the fill factor.

Torres et al. [129] used a three-stacked FDS bimorph to fabricate a large-angle micromirror device. Unlike conventional fabrication methods, achieving a high fill factor involved fabricating the actuator and mirror separately and subsequently bonding them together using silver epoxy. The designed mirror, as shown in Figure 7e–g, exhibits a TTP motion of $\pm 23^{\circ }$ and 127 μm, respectively. Recently, Xia et al. [137] demonstrated ISC bimorph-based new ETA shapes, termed as “O” and “Z” type ETAs. The new micromirror devices were designed to be used for fast scanning with a compact size in photoacoustic microscopy (PAM) systems. The proposed micromirror demonstrated significant achievements, with a piston movement 550μm and a scan angle ranging over $\pm 30^{\circ }$ under a 0–10 $V_{dc}$ excitation. It also exhibited a high resolution of up to 9 μm and achieved a scanning time of 12 seconds for a region measuring 1 mm × 4 mm.

Large vertical displacement (LVD) in micromirrors is desired in a number of applications. ETA-based micromirrors offer a good few hundred micrometers of LVD. However, during large piston/vertical displacement, the micromirrors also suffer from lateral shift, which in turn can lead to a distortion in imaging systems. For example, [138] offered an LVD of 710 μm with an undesired lateral shift of 420 μm. Hence, it is desirable to have minimum or no lateral shift. Lateral shift-free (LSF) actuation can be attained by using multiple segments of bimorphs. In this regard, Wu et al. [130] presented a three-bimorph actuation system with an aim to reduce the lateral shift to an insignificant level. The bimorphs, comprising Pt as heating material and driving a micromirror of size of 0.8 mm × 0.8 mm, attained a vertical displacement of 620 μm. The micromirror had a lateral shift of only 10 μm, which is negligible compared to the vertical displacement. The LSF concept using three bimorphs is shown in Figure 7h,i. Subsequently, the same group expanded upon this concept by introducing an array offering TTP movements [131]. The 4 micromirror array, as shown in Figure 7j, was capable of TTP motions and had a fill factor of 54%. An optical angle, defined as the deflected or redirected angle of a light beam of $\pm \;18^{\circ }$, was covered at 4.5 V, whereas a piston motion of 200 μm was attained.

For LSF, It is also required for the segments to have and equal change in temperature. However, by convection, different segments experience different temperature changes, which eventually cause a lateral shift to an extent. In 2022, Tang et al. [139] proposed a new bimorph design with varying segment widths to achieve uniform temperature changes across the actuator and ensure an LSF actuation.

The reliability issues associated with Al/$SiO_{2}$ ETA micromirrors were studied by Wang et al. [140]. A relatively low melting point of aluminum (600 °C) and brittleness of $SiO_{2}$ can lead to overheating and fractures in the bimorph [141]. To increase the reliability of the bimorph beam, Zhang et al. [142] proposed a Copper and Tungsten, Cu/W, bimorph ETA micromirror in 2015 and subsequently improved by the same group in 2019 [143]. The bimorph pair, having a high TEC difference, employed the ISCB principle for the formation of Cu/W bimorph and Cu/W/Cu multimorph beam. The fabricated MEMS mirror exhibited a piston displacement of approximately 114 μm and a tip–tilt optical angle of $\pm 8^{\circ }$ at 2.35 V. Pal et al. [144] introduced a novel bimorph design to address mechanical shocks, substituting $SiO_{2}$ with tungsten (W) and employing polyimide as the thermal isolator. The resulting devices demonstrated notably better robustness in comparison to previously documented mirrors utilizing Al-$SiO_{2}$ bimorphs and $SiO_{2}$ thermal isolation.

Recently, Yang et al. [145] reported a poly material, photosensitive polyimide (PSPI), reliable and LSF micromirror device. The PSPI serves to partially replace the $SiO_{2}$ at anchor points and for isolation, offering 4–5 times enhanced resistance to acceleration forces. Furthermore, the PSPI-based micromirror device attained a vertical displacement of 370 μm and an optical scan angle, defined as the total range of light deflection, of $\pm 19.6^{\circ }$ with just four $V_{dc}$.

## 4. Electromagnetic Actuation

Electromagnetically actuated micromirrors (EMAMs) typically consist of a magnetic coil attached to the micromirror, which is induced by a permanent magnet that is placed in the geometry of the mirror. A current flow in the coil generates a magnetic field, which interacts with the field of permanent magnetic to produce a repulsive or attractive force. Since the flow of the current direction can produce a magnetic field in both directions, the actuators can be pushed or pulled to move the mirror plate in a bidirectional direction through the line of action. This bidirectionality gives an advantage to EMAMs over other actuation techniques. However, the inclusion of coil structure and permanent magnet brings some complexity to their fabrication as well, in small size in particular. In this section, different design techniques employed to fabricate EMAMs for different applications will be discussed.

**Figure 8:**
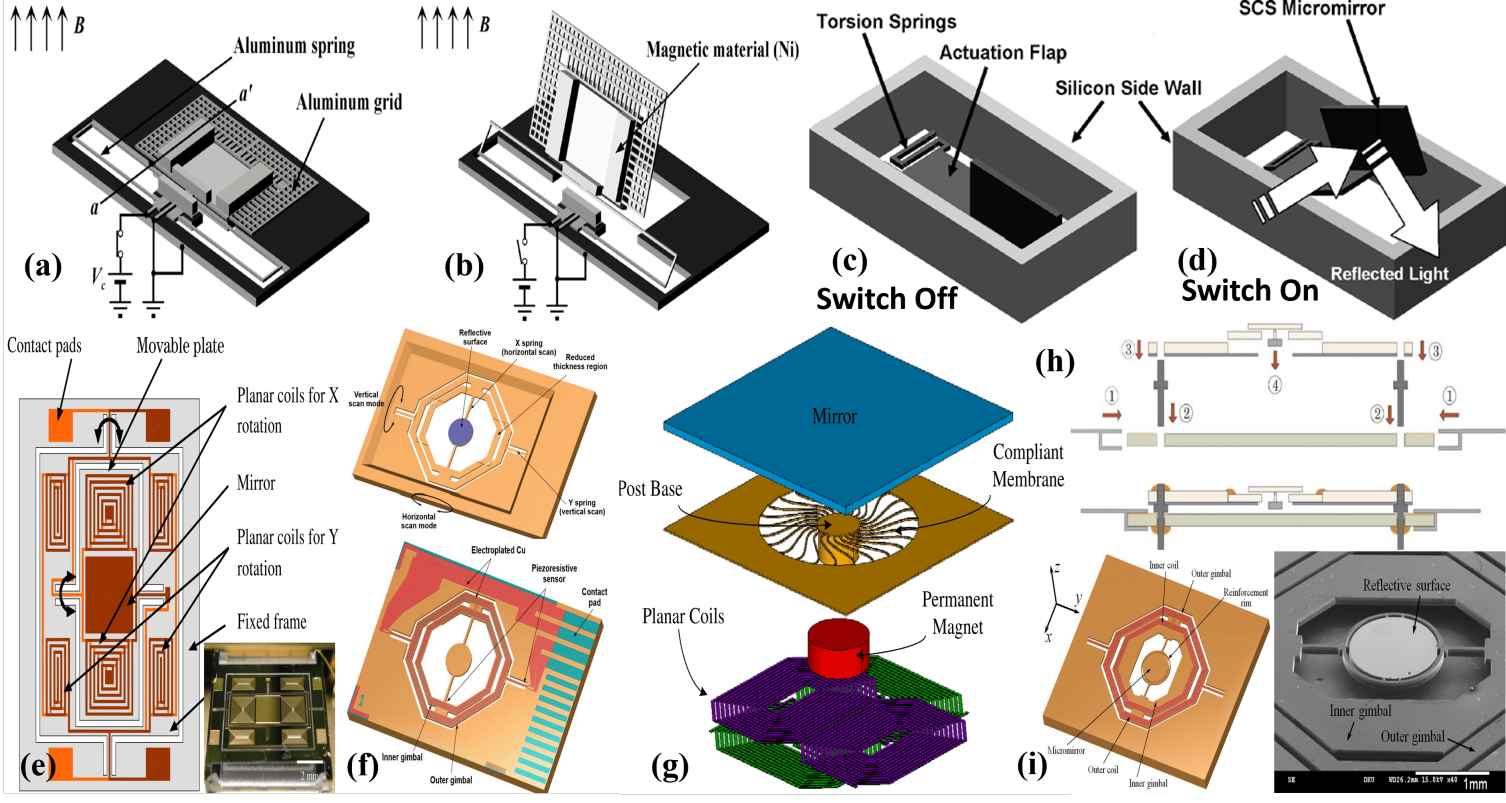
(**a**) Schematic of the aluminium spring based EMAM clamped under ES force. (**b**) Under electromagnetic actuation. Adapted with permission from Ref. [149]. Copyright 2003, IEEE. (**c**) Vertical EMAM in switched-off status. (**d**) Vertical EMAM when switched ON. Adapted with permission from Ref. [155]. Copyright 2005, IEEE. (**e**) Schematic of the optical scanner for OCT imaging with optical image of the fabricated EMAM. Adapted with permission from Ref. [156]. Copyright 2006, IOPScience. (**f**) Schematic of 2D double gimbal micromirror. Adapted with permission from Ref. [5]. Copyright 2018, IEEE. (**g**) Illustration of dual-axis micromirror moving-magnet actuation; (**h**) Integration of the sequence of the components (top); cross-section of the integrated system (bottom). Adapted with permission from Ref. [157]. Copyright 2012, IOPScience. (**i**) Design of the mirror with mechanical amplification (left); SEM image of the fabricated mirror (right). Adapted from Ref. [158]. Available under a CC-BY license. Copyright 2015, Optica.

One of the early EMAMs was developed by electroplating polysilicon and SCS moving parts with magnetic materials [146,147]. The 450 μm^2^ polysilicon mirror plate, patterned with nickel, was attached to the substrate with torsional beams. The torsional beams can be actuated by passing a current in the magnetic coil integrated into the substrate, generating a large displacement of up to 1 mm [146]. Polysilicon and SCS served as mirror plates and spring materials. However, polysilicon can present issues with surface roughness and stress, while controlling the thickness of SCS structures is challenging. These issues in surface micromachined polysilicon and crystal silicon mirrors pose significant concerns. In this regard, an EMAM with a mirror plate and torsion beam composed of electroplated nickel was proposed by Prasciolu et al. [148]. The torsion beam, carrying a nickel plate mirror of 1200×1400×4μm^3^, was connected to an electroplated copper made free standing structural frame. A 500-turn copper coil wrapped on a 7 cm iron rod, inducing the electromagnetic actuation, was integrated to the mirror frame. The EMAM is capable of generating a deflection of >24° in both directions. Another study employed making the mirror area using crystal silicon rather than nickel to increase the reflectivity [149]. The mirror was attached to Al springs and also surrounded by an Al grid, which acted as a clamping electrode. The clamping effect was provided by an electrostatic force to cancel out any force induced by an external magnetic field. The mirror shown in Figure 8a,b can rotate beyond $80^{\circ }$ when subjected to an external magnetic force. The same group extensively researched on EMAMs for different applications like optical switch [18], projection display [5,150,151,152,153], and LIDAR systems [14,154]. Su et al. [155] presented an SCS-based vertical EMAM for 2D optical switches matrix. A vertical mirror of the size of 500 × 1200 μm^2^ was shaped and smoothed by a wet etch process using tetra-methyl ammonium hydroxide as an etchant. The vertical micromirror, with in/out movement in the optical path—as shown in Figure 8c,d—capable of solving angular misalignment issues, was successfully utilized in a 10×10 array. Hwang et al. [14] presented a compact, low-power scanning micromirror. The mirror, having a reflective surface of 5 mm in diameter, can reach a $30^{\circ }$ torsional tilt when operated at 17.4 $mA_{rms}$, which corresponds to 7.8 $mW_{rms}$.

A number of 2D electromagnetic micromirrors were developed to perform biaxial motion to achieve image scanning for display systems [159,160,161,162,163]. In 2006, Mitsui et al. [156] presented a dual-axis EMAM for OCT imaging. The EMAM, shown in Figure 8e, consisted of planar coils positioned above a permanent magnet. Two coils were built forthe rotation of an internal mirror carrying plate in the X axis, whereas the Y axis rotation was induced by fabricating four coils on a movable frame. An optical scanning angle of $\pm 8^{\circ }$ can be generated with a drive current of ±4.6 mA and ±10.3 mA for the X and Y axes, respectively.

In 2018, Ju et al. [5] presented a biaxial electromagnetic scanning micromirror designed for projection display systems. A double-gimbal-structured actuator was used to drive a mirror with 1.2 mm diameter, as depicted in Figure 8f. The maximum vertical and horizontal scanning angles were $33^{\circ }$ and $52^{\circ }$, respectively. Later, from the same research group, Kang et al. [153] presented a 2-axis scanning EMAM with a 6.4 mm diameter. The fabricated micromirror demonstrated scanning angles of up to $25.6^{\circ }$ and $35.3^{\circ }$ on the vertical and horizontal axis, respectively. In a recent report, Zhou et al. [164] proposed a large-size 2D EMAM scanning micromirror with a magnetic field with a fixed angle of operation at $45^{\circ }$. The micromirror, with a reflective surface of 5 mm × 7 mm, prepared a 2D scanning EMAM with horizontal and vertical scanning angles of $10.4^{\circ }$ and $23.4^{\circ }$ at 5 V.

Until now, we have only discussed micromirrors with stationary magnets. Another approach is to place a moving magnet above a stationary coil-based biaxial EMAM, as reported by [157]. In this design, a moving mirror is attached to the magnet via a compliant membrane. All components are separately fabricated and then integrated together to achieve high yield. The moving parts containing the magnet-attached mirror do not require any electrical contact, which simplifies the design. The 4×4 mm^2^ micromirror, depicted in Figure 8g,h, can exhibit a mechanical rotation of $\pm 8.6^{\circ }$ on both axes when the coils are driven at 400 mA. Cho et al. [158] presented a mechanism to maximize the magnetic field strength. Copper coils were fabricated on two gimbaled scanners using CSC layers, as shown in Figure 8i. An optimized magnet array was placed under the substrate at a $45^{\circ }$ angle to maximize the strength. The final device, comprising a mirror with a 1.2 mm diameter, can scan $\pm 36.12^{\circ }$ on the horizontal axis and $\pm 17.62^{\circ }$ on the vertical axis when an input current of 515.17 mA is applied.

EMAMs generally consist of separate elements for mechanical support and electromagnetic actuation. Recently, CH.Ou et al. [165,166] presented a metallic glass-based electromagnetic actuator. The metallic glass not only possesses high elasticity but also integrates mechanical support and actuation elements into a single layer. The primary goal of the presented EMAMs was to achieve a large stroke. They have successfully demonstrated a significant upward static stroke of 418 μm.

**Figure 9:**
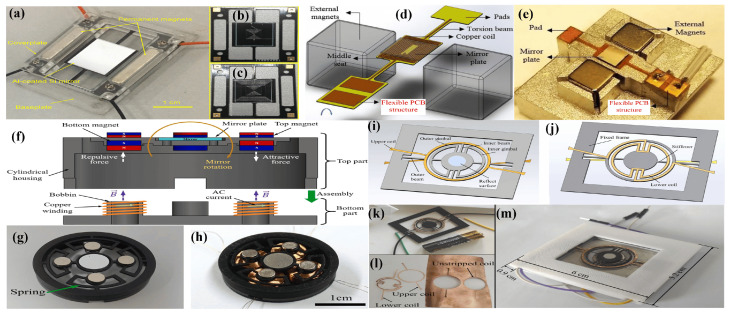
(**a**) Prototype of FR4-based scanning EMAM enclosed in a plexiglass package; (**b**) frontal copper coils for sensing; and (**c**) back-side of the FR4 platform for driving and sensing. Adapted from Ref. [167]. Available under a CC-BY license. Copyright 2018, MDPI. (**d**) Structure of flexible PCB EMAM and (**e**) the assembled flexible PCB. Adapted with permission from Ref. [169]. Copyright 2019, IOPScience. Shown are 3D-printed mechanical structures-based micromirrors (**f**) Operation principal of mirror described in [173]. (**g**) Structure with mirror and magnets. (**h**) Final assembled scanning device. Adapted with permission from Ref. [173]. Copyright 2022, Elsevier. (**i**) Upper coil assembly of the biaxial scanning mirror presented by Ref. [174]. (**j**) Lower coil assembly; (**k**) structure of the coil bonded mirror; (**l**) laser-patterned coils made of copper foil; (**m**) the final packaged mirror. Adapted with permission from Ref. [174]. Copyright 2022, Elsevier.

Printed circuit board (PCB) technology is used in magnetic micromirrors to fabricate large-aperture and low-cost scanning devices [167,168,169]. The employed PCB technology demonstrates an FR4-based EMAM, where copper coils are patterned on the surface of FR-4 [167,168]. Subsequently, an 11.6 mm Al-coated silicon mirror is bonded to the FR-4 substrate. Permanent magnets were positioned to generate a magnetic field parallel to the mirror. Along with the driving coil, a sensing coil has also been printed, which can track the real-time movement of the mirror plate. The position sensing mechanism for real-time tracking is based on a change in inductance when the plates are moving [170]. The assembled prototype, depicted in Figure 9a–c, achieved a $11.2^{\circ }$ of an optical scan angle at 425 mV. Flexible PCB structures with coils embedded in the polyimide layers were used to fabricate large aperture micromirrors [169,171,172]. The system consists of two polyimide torsion beams connected to the mirror plate, which sit in the middle of a structure with printed coils [169]. The beams are supported by two pads, whereas two permanent magnets are placed to generate a perpendicular magnetic field. The final device, shown in Figure 9d,e, with a mirror size of 4×4 mm^2^, can achieve a rotation angle of $13^{\circ }$ at ±400 mV driving voltage.

The use of 3D printing has also been employed in the fabrication of EMAMs [173]. Instead of using costly and time-consuming MEMS technology, 3D printing was adopted to fabricate an acrylonitrile butadiene styrene (ABS) polymer-based core assembly. The separately fabricated 6 mm mirror, coils, and magnets were then assembled into the core part and are depicted in Figure 9f–h. The device successfully obtained an optical scan angle of $8.98^{\circ }$ and $7.9^{\circ }$ on the horizontal and vertical axes, respectively, at 0.2 $A_{rms}$. Another research group demonstrated laser patterning along with 3D printing [174]. Like in [173], the mechanical structure of the scanning system was printed using ABS polymer, and then laser-patterned copper foil precise coils were assembled at the upper and lower sides of the structure, as shown in Figure 9i–l. The 10 mm mirror-based final package is shown in Figure 9m and can scan a vertical angle of $32.4^{\circ }$ and a horizontal angle of $16.5^{\circ }$ at 0.9 $A_{rms}$.

EM actuation has the advantage of having a better linear response in comparison to other techniques, making it a good candidate for linear deflection applications. The disadvantages associated with EMAMs include large thermal dissipation, the requirement of a permanent magnet, high power consumption, and large size taken due to an external magnet.

## 5. Piezoelectric Actuation

Piezoelectric actuators have also been employed for steering of MEMS micromirrors. The piezoelectric actuators used to drive these MEMS structures are the multilayer piezo unimorphs, which bend when a voltage is applied to it. Like electrothermal bimorphic beams, the piezo unimorph actuators are formed by sandwiching a piezoelectric material layer with other nonactive conductive material layers.

**Figure 10:**
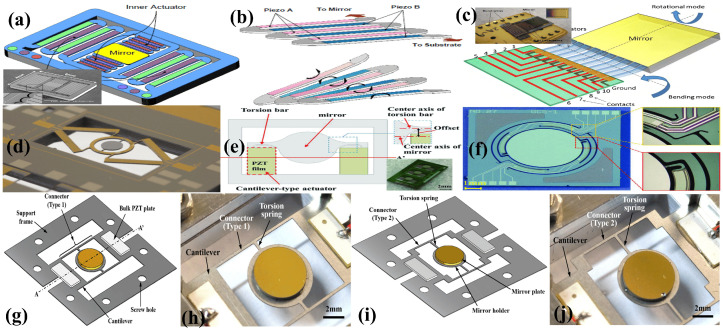
(**a**) Schematic of the 2D scanner with SEM image of the fabricated scanner. (**b**) Actuation mechanism. Adapted with permission from Ref. [176]. Copyright 2007, IEEE. (**c**) Schematic of MEMS scanner actuated by an array of PZT actuators and photograph of the packaged device. Adapted with permission from Ref. [177]. Copyright 2010, Elsevier. (**d**) Gimble-mounted piezoelectric 2D micromirrors. Adapted with permission from Ref. [179]. Copyright 2015, IEEE. (**e**) cantilever-type actuator based micromirror. Adapted with permission from Ref. [182]. Copyright 2024, IEEE. (**f**) Fabricated circular arc beam structured micromirror with a bell-shaped connection to the X axis actuator and U-shaped connection to the Y axis actuator. Adapted with permission from Ref. [183]. Copyright 2024, IEEE. Steel plate based 1D micromirror: (**g**) Schematic of structure #1. (**h**) Assembled structure #1. (**i**) Schematic of structure #2. (**j**) Assembled structure #2. Adapted with permission from Ref. [184]. Copyright 2024, Elsevier.

Like other MEMS-based micromirrors, piezoelectric mirrors (PEMs) are also capable of demonstrating 1D or 2D rotation. The rotation is achieved by torsional movement, bending, or a combination of both torsional and bending movements [175]. Typically, two actuators are positioned symmetrically on either side of the rotation axis to provide support for the torsion bars. In 2007, Tani et al. [176] proposed a unimorph piezoelectric actuator in a cascaded form for a 2D optical scanner. The cascaded cantilevers were formed by depositing lead–zirconate–titanate (PZT) film on a silicon substrate. The actuation mechanism and the schematic of the optical scanner are depicted in Figure 10a,b. Opposite polarities voltages were applied to the alternate arms of the cascaded cantilever to induce deflection in opposite directions, thus generating large scanning angles. The scanner, with a mirror of size 1.0 mm × 1.5 mm, demonstrated a mechanical angle of $\pm 8.6^{\circ }$ at 20 V. In 2010, Koh et al. [177] presented an arrayed PZT actuator consisting of 10 elements to drive the micromirrors, as depicted in Figure 10c. The notable aspect of the design is connecting the mirror only from one end, yet it is capable of producing biaxial movement. Two devices with mirror sizes of 5×5 mm^2^ and 3×3 mm^2^ were fabricated. At an applied voltage of 10 $V_{pp}$, an optical angle of $2.8^{\circ }$ has been achieved, whereas the device also can reach $0.56^{\circ }$ in torsional mode operation at 10 $V_{pp}$. In 2012, Baran et al. [6] presented a unimorph PZT MEMS mirror for display systems. The scanner, with a mirror size of 1.4 mm, can attain an optical scan angle of $38.5^{\circ }$ when actuated at 24 $V_{pp}$, while having a low power consumption of about 28 mW. Gu et al. [178,179,180] presented gimble-mounted piezoelectric 2D micromirrors for biaxial scanning. One such micromirror device is shown in Figure 10d, which exhibited a scan angle of $21.4^{\circ }$ and $31.3^{\circ }$ on the x axis and y axis, respectively. Recently, Vergara et al. [181] introduced a 1D piezoelectric micromirror designed for quasi-static actuation at low frequencies. The device incorporates embedded piezoresistive sensors to enable feedback control, thus attaining precise position control.

Though mirrors with symmetrically placed actuators across the axis offer stable rotation and high resonant frequencies, they often require high voltage due to the stiffness of their actuators. Recently, Abe et al. [182] proposed a cantilever-type actuator designed to offer a wide angle and low voltage operation. The mirror, depicted in Figure 10e, has a reflective region of 1 mm × 1.2 mm and has demonstrated an optical scan angle of around $89^{\circ }$ when actuated at $7\;V_{pp}$.

Although the PZT film is known for its exceptional piezoelectric properties, its complex fabrication process and incompatibility with integrated circuits (ICs) and MEMS limits its broader application. On the other hand, aluminum nitride (AlN) film emerges as a more promising material for piezoelectric micro-actuation applications, owing to its simple deposition process and excellent compatibility with IC and MEMS technologies. In 2018, Shao et al. [185] proposed the AlN film-based piezoelectrically actuated MEMS micromirror for the first time. Nevertheless, since AlN has a lower piezoelectric coefficient, despite having a small reflective surface of 200 μm × 200 μm, a small rotational angle of only $1^{\circ }$ was attained at 5 V. In the same year, Lei et al. [186] reported a piezoelectrically actuated micromirror with a leverage and resonant amplification mechanism to deal with the low piezoelectric coefficient issue of the AlN. A 6 mm × 4 mm mirror with angle monitor sensor was driven at 10 V to achieve a scan angle of $8.2^{\circ }$. Meinel et al. [187,188] reported an AlN-based micromirror with a tilt angle of up to $34.5^{\circ }$ (corresponding to $137.9^{\circ }$ optical scan) when actuated at 20 V. A mirror with 800 μm × 800 μm reflective plate had a voltage sensitivity of 4.7°/V.

The same research group, in 2022, presented statically actuated high-voltage micromirrors based on AlN and aluminum scandium nitride (AlScN) [189]. For the same size of devices, the use of AlScN elevated the maximum scan angle from $8.4^{\circ }$ for AlN-based micromirrors to $38.4^{\circ }$ for AlScN-based micromirrors. Furthermore, AlScN also has been shown to have a higher electric breakdown voltage with a maximum actuation voltage of around 400 V compared to AlN-based actuators with 200 V. In a most recent article, Huang et al. [183] presented an AlScN-based biaxial micromirror with a circular arc beam structure. The circular arc beam is capable of attaining a large scanning angle. The micromirror, depicted in Figure 10f, with a 5 mm diameter, can achieve an optical scan angle of $25^{\circ }\times 22^{\circ }$ on the horizontal and vertical axes, respectively. The scanning range can be elevated to $52^{\circ }\times 42^{\circ }$ if the system is operated in a vacuum.

Recently, an unconventional method was adopted for the fabrication of a micromirror using a stainless steel plate for the structural layer instead of SCS substrate [184]. Two different devices with distinct geometries were modeled and experimentally assessed. The fabricated devices utilized PZT plates as actuating material, which were placed symmetrically on opposite sides of the mirror. A 5 mm silicon plate, coated with gold, was affixed to the mirror-holding assembly to act as the reflective surface, as depicted in Figure 10g–j. One device, referred as model #1 in Figure 10g–j, attained a scan angle of 26°, whereas model #2 achieved a scan angle up to 44° with an applied voltage of $200\;V_{pp}$.

## 6. Hybrid Actuators Based Micromirrors

As discussed, each actuation mechanism has its distinct advantages and limitations. However, some disadvantages limit the performance, functionality, and reliability of signal actuation techniques for a particular application. In this regard, different hybrid actuated micromirrors have been reported to combine the advantages of different actuation methods to minimize the limitations and enhance performance.

**Figure 11:**
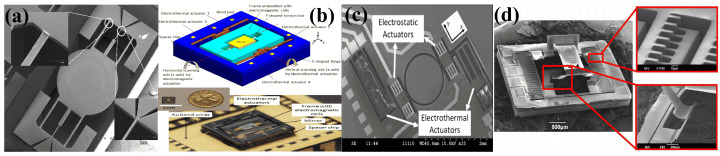
Hybrid electrothermal–electromagnetic micromirrors: (**a**) Hybrid mirror with zoomed images of buckle beams and torsion bar. Adapted with permission from Ref. [191]. Copyright 2009, IOPScience. (**b**) Schematic and optical image hybrid micromirror proposed by [192]. Adapted with permission, Copyright 2012, IEEE. Hybrid electrothermal–electrostatic micromirrors: (**c**) Presented by Ref. [193] and adapted with permission, Copyright 2011, IEEE. (**d**) Presented by Ref. [194] and adapted with permission, Copyright 2013, IEEE.

In 2009, Eun et al. [190,191] presented an electrothermal and electromagnetic actuators-based hybrid micromirror. The mirror, fabricated on an SOI wafer, has four buckle beams placed adjacent to the torsional beam. The mirror can be employed in both resonant and static modes. Electrothermal actuators provide the driving force in static mode operation, while the Lorentz force governs the direction of thermal buckle beam bending. Whereas during resonant mode operation, the electromagnetic force is used for both driving and directing of the reflective plate. The micromirror with a 1 mm reflective plate, shown in Figure 11a, covers an optical angle of around $27^{\circ }$ at $7.5\;V_{dc}$ and an optical angle of $17^{\circ }$ at resonance (10.64 kHz) at up to $4.4\;V_{pp}$.

In 2012, Koh et al. [192,195,196] proposed a 2D scanning mirror based on electromagnetic and electrothermal actuators to scan the slow and fast angles, respectively, as depicted in Figure 11b. The electrothermal actuator, composed of depositing Al on a silicon beam, presents a scanning angle of $\pm 1.5^{\circ }$ with 12 mW power consumption at 74 Hz. In contrast, the electromagnetic actuator governs the horizontal scan angle, achieving $\pm 10^{\circ }$ at 1.26 mA and 102 Hz when operated at 1 Vac. Electromagnetic actuators are also integrated with piezoelectric micromirrors to calculate the deflection of the mirror [197].

Electromagnetic actuators were also combined with electrostatic actuators to increase tilt angles while keeping the voltage and power requirements within a limit. In 2018, Alneamy et al. [198] proposed an EM- and ESA-based a hybrid micromirror, where electromagnetic actuators were used for tilt actuation, while ESA performed piston motion independently or in combination with the electromagnetic actuator.

Hybrid micromirrors based on electrothermal–electrostatic actuation have also been proposed [193,199,200]. Li et al. [193] developed a hybrid micromirror using SOI technology and based on their previous design reported in [200], which is depicted in Figure 11c. The outer thermal actuators scanning vertically achieved a scan angle of $10^{\circ }$ at 15 V, while the inner electrostatic actuators produced a $24^{\circ }$ horizontal scan angle at a resonant frequency of 1580 Hz. Another group also proposed electrothermal–electrostatic-based hybrid micromirrors [194,201]. In the first report [194], the presented micromirrors depicted in Figure 11d could produce significant in-plane and out-of-plane displacements. The out-of-plane movement was facilitated by a pair of bimorphic ETAs, reaching up to 370 μm with just 2.5 V. However, a negligible in-plane displacement was achieved using electrostatic CDA. Later in 2016, they modified the design to attain 40 μm in-plane displacement at $109.6\;V_{dc}$ for the electrostatic actuator and 100 μm out-of-plane displacement induced by the bimorph actuators at just $13.5\;V_{dc}$.

## 7. Discussion

In this paper, we discussed different actuation methods being used in MEMS micromirror technology. Due to their small size and lower cost, MEMS mirrors play a crucial role in different scanning and biomedical applications where compactness is desired. In addition to size, other important parameters of concern for MEMS micromirrors include the scan angle and scan speed. Ideally, a mirror should have an appropriate size, a large field of view, and high speed for better scanning and imaging results.

All the four types of actuation techniques used in micromirrors, as depicted in Figure 1, have been considered. The key characteristics of these differently actuated micromirrors are summarized in Table 1. Across the actuation methods, the sizes of the reflective surface of the micromirror devices varied from as small as 10 μm to as large as 10 mm. Electrostatically actuated micromirrors are generally chosen for their smaller sizes, whereas electromagnetic mirrors have the largest reflective surfaces. Nevertheless, the average size realized by most of the reports is around 1 mm.

### 7.1. Key Characteristics

The stroke induced by different actuation methods is analyzed. Parallel plates electrostatic actuators induced a maximum tilt angle of $12^{\circ }$ for a 25 × 25 μm^2^ mirror, whereas combdrive actuators achieved $15^{\circ }$ for a 1500 × 1500 μm mirror, with a slight difference in the operating voltage. However, the maximum tilt angle achieved by a combdrive actuator is $27^{\circ }$ for a 650 × 650 μm. On the other hand, parallel plate actuators performed better than the combdrive for piston motion, achieving 10 μm compared to 1.2 μm. Electrothermal actuators with an average mirror size of 1 mm have an average tilt angle of around $30^{\circ }$ and piston motion of >100 μm, with the maximum reported optical scanning angle and piston motion being $124^{\circ }$ and 620 μm, respectively. Electromagnetic actuator micromirrors with a large reflecting area have an average tilt angle of $20^{\circ }$ with a maximum of $32^{\circ }$ for a device with a mirror size of 10 mm. Lastly, piezoelectric actuated micromirrors have induced an angular motion of $35^{\circ }$ for a mirror sized 800 × 800 μm^2^ when a voltage of 20 V is applied.

### 7.2. Advantages and Trade-Offs

There are several advantages and disadvantages associated with every actuation technique. The advantages and drawbacks of MEMS micromirror actuation methods are summarized in Table 2 based on [30]. The advantages of electrostatic actuation include fast response, low power consumption, and no heat dissipation. However, the key drawbacks are high voltage requirements, pull-in effect, and nonlinear response. ESAs can be efficiently used to steer mirrors with relatively smaller sizes (around 2 mm) with a small air gap between the plates. Otherwise, the driving voltage will drastically increase, making it impractical. Electrothermal actuators offer several advantages compared to the other actuation techniques. They operate at lower voltages, provide large angular and piston motion, and do not have electromagnetic interference or electrostatic discharge issues. However, they have slow response times, high power consumption, and heat dissipation issues. The heat dissipation can affact operational conditions and can change the thermal properties of the moving beams, making the control of movements challenging. Electromagnetic actuators exhibit large angular deflection, better linear response, and low operational voltage. On the other hand, they require external magnet and the deposition of coils in a large-sized assembly. Furthermore, they have high power consumption and significant thermal dissipation. Finally, the piezoelectric actuators offer the advantages of low driving voltage and low power consumption. However, due to complexity of depositing piezoelectric material layers, the fabrication is complex, and they also have a large footprint with a relatively small reflective surface.

The advantages and drawbacks of each actuation method do not make them inherently good or bad; rather, they provide a range of options for different applications. The suitability of an actuation method of a micromirror vary significantly based on the application. Critical parameters such as stroke, mirror area, and rotation angle must be carefully considered. For instance, in handheld or wearable devices, where low power consumption and compact design are crucial, a micromirror that operates at low voltage would be most suitable. In such cases, electrothermal or electromagnetic actuation would offer advantages due to their ability to provide larger strokes at lower voltages, this may lead to some trade-offs in terms of response speed, rotational angle, and heat dissipation. On the other hand, in applications requiring high-speed operation and rapid switching, such as display projections or optical switching, electrostatically actuated mirrors would be preferable due to their fast response times and lower power requirements, even though they may require higher voltages and are prone to pull-in effects. In terms of specific performance characteristics, the stroke length can be crucial for applications requiring larger deflections, such as endoscopic imaging, whereas the rotation angle is a key factor in scanning applications like LiDAR, where a wider field of view is desirable. For display applications, the mirror’s ability to maintain beam quality under rapid actuation is essential, favoring designs that minimize deformation, even if this means accepting a higher driving voltage or reduced scan speed. Ultimately, the selection of an actuation technique should be based on a clear understanding of these performance trade-offs, allowing scientists and engineers to select a model that best aligns with the desired application. By considering the importance of stroke, rotation angle, power consumption, and response speed, a micromirror design and actuation method can be chosen to meet specific requirements.

### 7.3. Limitations and Research Gap

MEMS micromirrors are used as an alternative to the conventional galvanometer mirrors, particularly in medical applications where compactness is a key factor. Although micromirrors have the advantages of small size and lower cost over galvanometer mirrors. However, galvanometer mirrors demonstrate better motion and position control over MEMS micromirrors due to their operation under the closed loop feedback system.

One of the major limitations of the MEMS micromirrors is the lack of control over their movement and real-time position sensing. The absence of a closed-loop feedback system makes it challenging to operate the micromirror linearly. Several efforts have been made to introduce a feedback and real-time position sensing mechanism into electromagnetic micromirrors [19,202,203,204]; however, these advancements have yet to be widely applied to other types of micromirrors. One possible solution could be the control mechanism of electromagnetic micromirrors into other types of micromirror actuation techniques.

Another issue with the micromirrors is surface deformation after actuation. Deformation can cause the mirror surface to form a curvature, which can contract or expand the reflected beam and may lead to loss of information in the reflected beam. The deformation issue can be addressed by using stiff mirror plate with elevated thickness. However, increasing the mirror thickness will affect the scanning angle. Therefore, the thickness should be optimized as per desired angular movement.

### 7.4. Validation and Sensitivity Analysis

**Figure 12:**
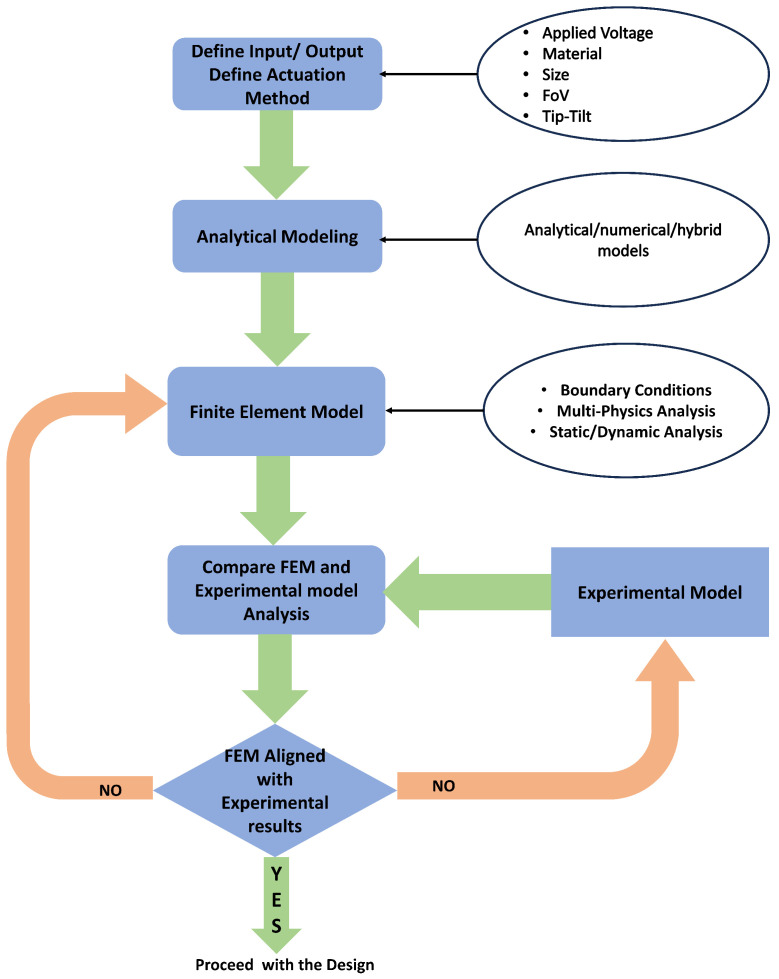
Design and validation methodology for MEMS micromirrors.

To ensure the reliability and accuracy of the micromirror actuation techniques discussed, it is crucial to validate these methods by studying the reliability and performance. Early prediction of the performance of these actuation methods can greatly influence design costs and enhance the overall quality. Incorporating model validation techniques into design methodologies is important for forecasting the micromirror’s performance. The validation process involves assessing how variations in parameters such as electrode gap, actuation voltage, and material properties affect the performance and predictions of the models. Several studies have been reported to validate the model, predict performance, study parametric uncertainities, and analyze the reliability of the miromirrors [205,206,207,208]. We will provide a brief overview rather than an in-depth discussion. A flowchart of the modeling and validation process is shown as Figure 12. The flowchart illustrates the steps involved in parameter selection, model validation, and the potential interactions between various parameters. The first critical step is to define the system’s inputs and outputs, along with selecting an actuation technique. Next, an analytical model is crafted to obtain initial values. These analytical results can then be integrated into a finite element model (FEM) for parameter optimization. The predictions generated by the FEM can be compared with the results from the experimental setup. If the experimental results align with both the FEM and analytical outcomes, the model can be adopted. However, if discrepancies arise between the modeled and experimental results, the FEM parameters will need to be revisited. If corrections are made, the experimental setup can then be improved. This visual representation offers valuable insights into the modeling and validation of performance parameters. Such analysis is essential for understanding the robustness of the models and ensuring they accurately reflect the real-world behavior of micromirrors.

### 7.5. Summary

MEMS micromirrors offer numerous advantages such as compact size, high speed, and low cost. Parameters like mirror size, scanning angle, speed, resonance, and power consumption play key roles in micromirrors. A careful consideration of these parameters makes MEMS mirrors an ideal candidate for applications in many fields, such as medical imaging, LiDAR, and projection displays. Their ongoing development and integration of advanced control mechanisms will likely enhance their performance and broaden their application scope in the future.

## 8. Conclusions

In this review, various actuation methods employed in MEMS micromirrors have been briefly explored. Mainly four actuation methods, including electrostatic, electrothermal, electromagnetic, and piezoelectric, have been utilized. Each actuation mechanism has some advantages and shortcomings compared to others. The key performance parameters of interest include scanning angle, response time, power consumption, and mechanical robustness. These parameters define the suitability of a micromirror for a specific application and plays an important role in choosing a particular actuation method. Electrostatic actuation stands out for its low power consumption and fast response, but it requires high operating voltages and can suffer from instability issues. Electrothermal actuation offers large deflection angles and strong driving force at low voltages but at the cost of higher power consumption and slower response times. Electromagnetic actuation provides high-speed scanning and large deflections but requires bulky external magnets and incurs higher power usage. Piezoelectric actuation balances moderate power consumption with relatively high driving voltages and complex fabrication processes. Hybrid micromirrors, combining multiple actuation techniques, is a promising approach to take advantage of the strengths of different methods while mitigating their weaknesses. Additionally, addressing challenges such as real-time position sensing and surface deformation is crucial for advancing the precision and reliability of MEMS micromirrors. Overall, MEMS micromirrors’ compact size, versatility, and cost-effectiveness make them ideal candidates for endoscopic imaging, LiDAR, display projections, and optical communication applications. Continued advancements in actuation techniques and control mechanisms will further enhance their performance, opening new possibilities for their use in various cutting-edge technologies.

## Figures and Tables

**Table 1:** Actuator techniques for MEMS micromirrors.

Actuation	Mirror Type	Material	Voltage/Power	Stroke	Size	Ref
Electrostatic	1D	nickel	35 V	10 μm	10 μm × 10 μm	[41]
Parallel plate	1D	Al	14 V	0.3 μm	30 μm × 40 μm	[42]
	2D	polysilicon	100 V	1.65 μm	400 × 400 μm^2^	[62]
	2D	polysilicon	150 V	5 μm	400 × 400 μm^2^	[47]
	2D	Al	36.5 V	$\pm 12^{\circ }$	25 × 25 μm^2^	[45]
	TTP	Au	23.1 V	1.5 μm	200 × 200 μm^2^	[21]
ESA	2D	polysilicon	48 V	$\pm 1.8^{\circ }$ & $\pm 2.1^{\circ }$	1 mm^2^	[71]
Combdrive	2D	epitaxial Si	40 V	$\pm 15^{\circ }$	1.5 × 1.5 mm^2^	[74]
	1D	polysilicon	100 V	$\pm 1^{\circ }$	450 μm	[70]
	2D	polysilicon	$20\;V_{dc}\;\&\;25\;V_{pp}$	$27^{\circ }$ & $20^{\circ }$	650 × 650 μm^2^	[75]
	2D	Si repulsive	60 V	1.2 μm	190 × 190 μm^2^	[96]
	1D	CNTs	5 V	$1.5^{\circ }$	–	[94]
	2D	Si repulsive	150 V	$11.5^{\circ }$	1 mm	[60]
Electrothermal	1D	polysilicon-$SiO_{2}$/Al	4.8 mW	$4.8^{\circ }$	–	[118]
	1D	polysilicon-$SiO_{2}$/Al	10 mW	$17^{\circ }$	–	[109,119]
	2D	polysilicon-$SiO_{2}$/Al	95 mW	$40^{\circ }$	–	[110]
	TTP	polysilicon-$SiO_{2}$/Al	15 V	$\pm 30^{\circ }\;\&\;500$ μm	–	[111]
	1D	Platinum-$SiO_{2}$/Al	12.5 V	$124^{\circ }$	–	[120]
	2D	Al/W	0.68 V/11mW	$60^{\circ }$	1 mm	[124]
	TTP	Al/W	0.6 V & 0.8 V	$\pm \;11^{\circ }$ & 227 μm	1 mm	[125]
	Piston	Al/W	0.9 V	200 μm	1 mm	[125]
	TTP	Pt-$SiO_{2}$/Al (FDS)	8 V	$\pm 30^{\circ }\;\&\;480$ μm	–	[127]
	Piston	Pt-$SiO_{2}$/Al (FDS)	1.2 V	90 μm	–	[134]
	2D	Pt-$SiO_{2}$/Al	5.5 V	$\pm 40^{\circ }$	0.72 mm × 0.72 mm	[135]
	TTP	$SiO_{2}$/Al	10 V	$\pm 30^{\circ }\;\&\;550$ μm	1 mm	[137]
	Piston	Pt (LSF)	5.3 V	620 μm	0.8 mm × 0.8 mm	[130]
	TTP	Cu/W (ISCB)	2.35 V	$\pm 8^{\circ }$ & 114 μm	1 mm	[143]
	TTP	Al/PSPI	4 V	$\pm 19.6^{\circ }\;\&\;370$ μm	1 mm × 1 mm	[145]
Electromagnetic	1D	polysilicon/nickel	–	1mm	450 μm^2^	[146]
	1D	nickel	–	$>24^{\circ }$	1200 × 1400 × 4 μm^3^	[148]
	1D	–	–	$80^{\circ }$	–	[149]
	2D	–	–	–	500 × 1200 μm^2^	[155]
	1D	–	17.4 $mA_{rms}$	$30^{\circ }$	5mm	[14]
	2D	–	±4.6 mA and ±10.3 mA	$\pm 8^{\circ }$	–	[156]
	2D	–	–	$33^{\circ }$ & $52^{\circ }$	1.2 mm	[5]
	2D	–	–	$25.6^{\circ }$ & $35.3^{\circ }$	6.4 mm	[153]
	2D	–	5 V	$10.4^{\circ }$ & $23.4^{\circ }$	5 × 7 mm^2^	[164]
	2D	–	400 mA	$\pm 8.6^{\circ }$	4×4 mm^2^	[157]
	2D	–	515.17 mA	$\pm 36.12^{\circ }$ & $\pm 17.62^{\circ }$	1.2 mm	[158]
	2D	FR-4/Al	425 mV	$11.2^{\circ }$	11.6 mm	[167]
	2D	Polyimide	±400 mV	$13^{\circ }$	4 × 4 mm^2^	[169]
	2D	ABS polymer	0.2 $A_{rms}$	$8.98^{\circ }$ & $7.9^{\circ }$	6 mm	[173]
	2D	ABS polymer/Cu	0.9 $A_{rms}$	$32.4^{\circ }$ & $16.5^{\circ }$	10 mm	[174]
Piezoelectric	2D	PZT	20 V	$\pm 8.6^{\circ }$	1.0 mm × 1.5 mm	[176]
	2D	PZT	10 $V_{pp}$	$2.8^{\circ }$ & $0.56^{\circ }$	3 mm × 3 mm	[177]
	1D	AlN	5 V	$1^{\circ }$	200 μm × 200 μm	[185]
	1D	AlN	10 V	$8.2^{\circ }$	6 mm × 4 mm	[186]
	1D	AlN	20 V	$34.5^{\circ }$	0.8 mm × 0.8 mm	[188]
	1D	PZT & steel plate	$200\;V_{pp}$	$44^{\circ }$	5 mm	[184]

**Table 2:** Advantages and disadvantages of different actuation methods as discussed by [30].

Actuation Method	Advantages	Disadvantages
Electrostatic	Fast response	High driving voltage
	Low power consumption	Low drive force
	No heat dissipation	Pull-in effect
	No thermal effect	Nonlinear response
Electrothermal	Low driving voltage	High power
	Large scan angle	Heat generation
	High fill factor	Slow response
		High power consumption
Electromagnetic	Large scan angle	Large size
	Low driving voltage	High power
	Large drive force	Need external magnet
	Better linear response	Large size
		Complicated fabrication
		Large thermal dissipation
Piezoelectric	Fast response times	Small reflection surface
	Low driving voltage	Complex fabrication
	Low power consumption	Large foot print
